# Supplementary material for: Reconciling Mining with the Conservation of Cave Biodiversity: A Quantitative Baseline to Help Establish Conservation Priorities
Source: PLoS One. 2016 Dec 20;11(12):e0168348. doi: 10.1371/journal.pone.0168348 (PMC5173368; doi:10.1371/journal.pone.0168348)
Supplement: S1 Dataset — (ZIP) [file pone.0168348.s002.zip › Taxa/Serra Norte/SN_2013/N5S/Lista N5S-77.pdf]

CAVIDADE N5S-0077

Classe	Ordem	Fam/Outros	Gên/Outros	Espécie	S-U	U-S	Zon.
Arachnida	Araneae	Pholcidae		jovem	X	X	E
Arachnida	Araneae	Salticidae		sp.2	X	X	E
Arachnida	Araneae	Theridiidae	*Echinotheridion*	sp.1	X		E
Arachnida	Araneae	Theridiidae	*Theridion*	sp.2		X	E
Arachnida	Araneae	Theridiosomatidae		jovem	X		E
Arachnida	Palpigradi	Eukoeneniidae	*Eukoenenia*	sp.1		X	E
Entognatha	Collembola	Entomobryidae		sp.4		X	E
Entognatha	Collembola	Paronellidae		sp.1		X	E
Insecta	Coleoptera	Scydmaenidae		sp.1		X	E
Insecta	Diptera	Chironomidae		sp.	X		E
Insecta	Heteroptera	Reduviidae	*Zelurus*	sp.	1	7	E
Insecta	Hymenoptera	Formicidae	*Camponotus*	sp.1	X		E
Insecta	Hymenoptera	Formicidae	*Crematogaster*	sp.1	X		E
Insecta	Hymenoptera	Formicidae	*Pachycondyla*	sp.1		X	E
Insecta	Hymenoptera	Formicidae	*Pheidole*	sp.2		X	E
Insecta	Hymenoptera	Formicidae	*Solenopsis*	sp.1		X	E
Insecta	Hymenoptera	Vespidae		sp.2	X		E
Insecta	Hymenoptera	Vespidae		sp.3		X	E
Insecta	Isoptera	Termitidae	*Nasutitermes*	sp.		X	E
Insecta	Lepidoptera	Noctuoidea		sp.5	1		E
Insecta	Lepidoptera			jovem		X	E
Insecta	Orthoptera	Gryllidae	Nemobiinae	jovem		1	E
Insecta	Psocoptera	Manicapsocidae	*Nothoentomum*	sp.1		X	E
Insecta	Psocoptera	Psyllipsocidae	*Psyllipsocus*	sp.4		X	E
Malacostraca	Isopoda	Dubioniscidae		sp.1		X	E
Malacostraca	Isopoda	Philosciidae		sp.1		X	E
